# Supplementary material for: Novel Conjugated Polymers Containing 3-(2-Octyldodecyl)thieno[3,2-b]thiophene as a π-Bridge for Organic Photovoltaic Applications
Source: Polymers (Basel). 2020 Sep 17;12(9):2121. doi: 10.3390/polym12092121 (PMC7570215; doi:10.3390/polym12092121)
Supplement: Supplementary file 1 [file polymers-12-02121-s001.zip › polymers-918412-supplementary.docx]

Supplementary Information

Novel Conjugated Polymers Containing 3-(2-Octyldodecyl)thieno[3,2-*b*]thiophene as a π-Bridge for Organic Photovoltaic Applications

Jong-Woon Ha, Jong Baek Park, Hea Jung Park and Do-Hoon Hwang*

1. Department of Chemistry and Chemistry Institute for Functional Materials, Pusan National University, Busan 46241, Republic of Korea; [newhju@naver.com](javascript:void(0);) (J.-W.H.), [magician_p@naver.com](javascript:void(0);) (J.B.P.), [heajungp@pusan.ac.kr](javascript:void(0);) (H.J.P.)

***** Correspondence: dohoonhwang@pusan.ac.kr; Tel.: +82-51-510-2232.

**Keywords:** organic photovoltaic; 3-(2-Octyldodecyl)thieno[3,2-*b*]thiophen; fullerene; high crystallinity

1. Measurements

^1^H and ^13^C nuclear magnetic resonance (NMR) spectra were measured on a Varian Mercury Plus 300 MHz spectrometer. Thermal analyses were carried out on a TA Instruments Q600 system under an inert N_2_ atmosphere with heating and cooling rates of 10 °C min^−1^. The UV−Vis absorption spectra were measured on a UV-1800 UV-Vis spectrophotometer. Gel permeation chromatography (GPC) was performed to determine the molecular weight and confirm the polydispersity indices (*PDI*s) using a Waters high-pressure GPC assembly (Model M590). Cyclic voltammetry experiments were performed with a CH Instruments electrochemical analyzer in acetonitrile solutions containing 0.1 M tetrabutylammonium tetrafluoroborate (Bu_4_NBF_4_) as the supporting electrolyte, with Ag/AgNO_3_ as the reference electrode, a platinum wire as the counter electrode, and a platinum working electrode. Density functional theory (DFT) calculations were performed using the Gaussian 09W package with the Becke three-parameter Lee−Yang−Parr (B3LYP) function and the 6-31G(d) basis set to elucidate the HOMO and LUMO energy levels and backbone planarity. 2D GIWAXS experiments were performed at the 3C beamlines of the Pohang Accelerator Laboratory (PAL), Korea. The X-ray had a wavelength of 1.2301 Å (10.0791 keV), and the incidence angle (-0.10°) was chosen to allow complete X-ray penetration in the polymer film. The surface morphologies of the polymer thin films were characterized using AFM (SPM System) operated in the tapping mode and transmission electron microscopy (TEM). The OPV performances were confirmed using a McScience K201 LAB50 Solar Simulator. The current density-voltage (*J-V*) curves of the devices were measured under AM 1.5G condition, and the light intensity was calibrated using a Si standard reference device (PV Measurements Inc., calibrated at the National Renewable Energy Laboratory). The external quantum efficiency (EQE) data were measured using a McScience K3100 EQX system. The EQE values were obtained as a function of wavelength in the range from 300 to 1100 nm using a Xenon Short Arc Lamp as the light source, and the calibration was performed using a Si photodiode.

2. Device Fabrication and Characterization

The inverted single-layer photovoltaic devices had the structure of indium-tin oxide (ITO)/ZnO/polymer:PC_71_BM/MoO_3_/Ag. The active layer solution was prepared in chlorobenzene (CB) (1.0:2.0 w/w, total concentration = 30 mg·mL^-1^) and stirred overnight at 80 °C. The effective area of the device was 9 mm^2^. ITO substrates were pre-cleaned *via* conventional methods and treated with UV-ozone plasma for 20 min. The ZnO layer as the electron transfer layer was deposited onto the ITO at 2,000 rpm for 30 s by spin coating, followed by baking at 200 °C for 60 min. The active layer was fabricated by spin coating at various rpm for 30 s in a glove box filled with argon. After drying the active layer, MoO_3_ (~8 nm) and Ag (~100 nm) layers were formed by thermal evaporation under a base pressure of 10^-7^ torr.

The structure of the hole-only devices was ITO/PEDOT:PSS/polymer:PC_71_BM/Au. The PEODOT:PSS layer (~30 nm) was fabricated by spin coating onto ITO glass (4000 rpm for 30 s) and then baking at 150 °C for 10 min. The blended solution of polymer:PC_71_BM in CB with DIO was spin coated onto the PEDOT:PSS layer. After drying the active layer, Au (~100 nm) was formed by thermal evaporation under a base pressure of 10^-7^ Torr. The *J–V* curves were measured using a computerized Keithley 2400 Source Meter in the dark.

**Scheme S1.** Synthetic routes for 3-alkylthieno[3,2-b]thiophene using Ni or Pd as catalyst.


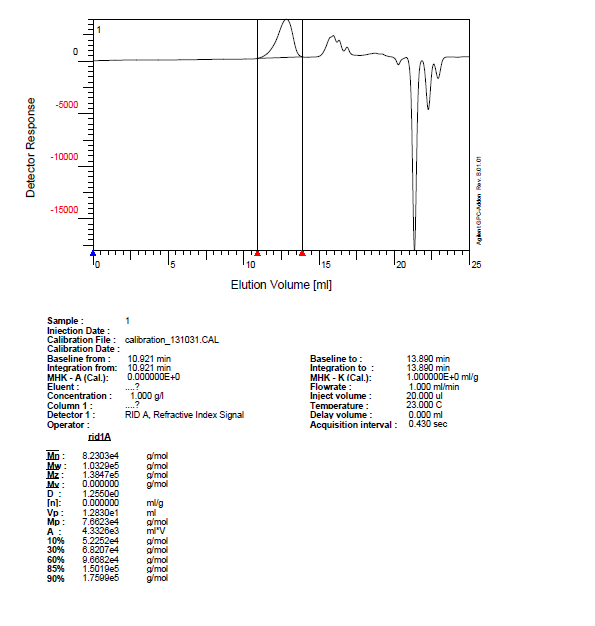


**Figure S1.** GPC data for PT-ODTTBT.

**
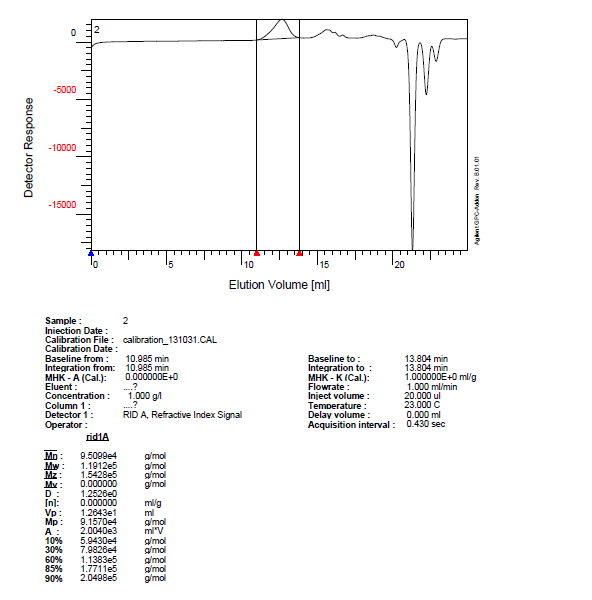
Figure S2.** GPC data for PTT-ODTTBT.


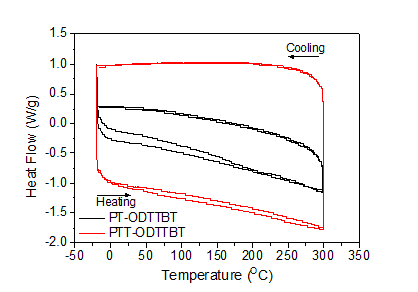

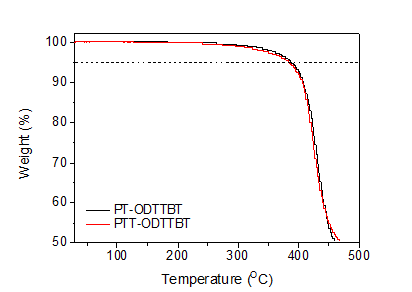


**Figure S3.** TGA (up) and DSC (down) curves of synthesized polymers.


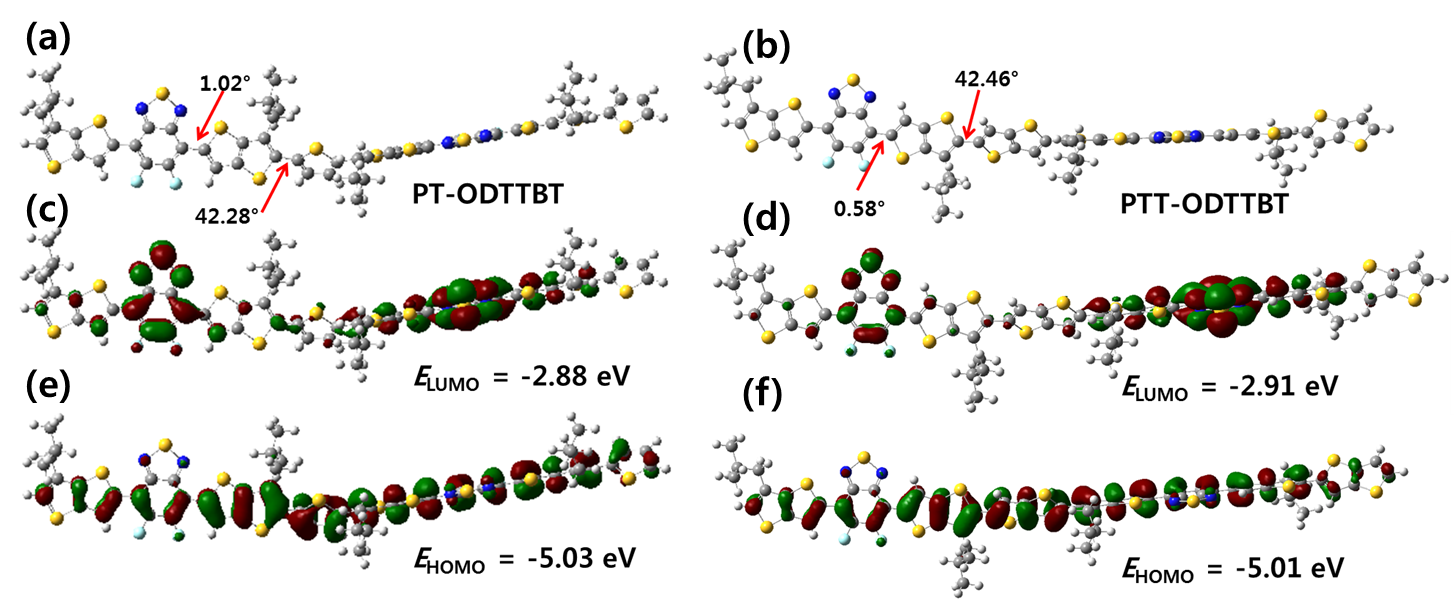


**Figure S4.** Optimized molecular models for (a) PT-ODTTBT and (b) PTT-ODTTBT. Energy levels and distribution of the corresponding (c, d) LUMO and (e, f) HOMO.

**Figure S5.** Cyclic voltammograms of synthesized polymers.


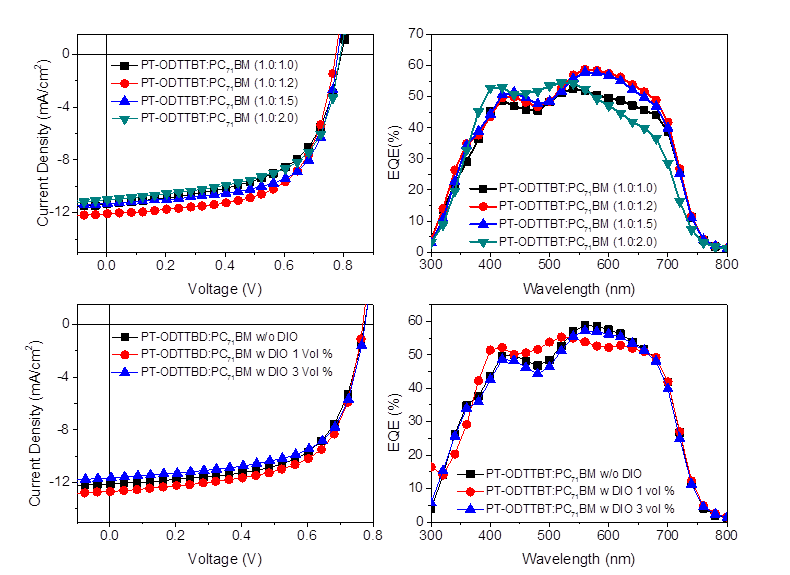


**Figure S6.** *J-V* characteristics and EQE curves of the OPV devices based on PT-ODTTBT:PC_71_BM with various blending ratios (up) and DIO volumes (down).

**Table S1.** Performances of the OPV devices based on PT-ODTTBT:PC_71_BM.

| **Ratio** | **DIO** | ***V*_oc_**  **(V)** | ***J*_sc_**  **(mA/cm^2^)** | ***J*_sc,calc._**  **(mA/cm^2^)** | ***FF***  **(%)** | ***PCE***  **(%)** |
| --- | --- | --- | --- | --- | --- | --- |
| 1.0:1.0 | - | 0.79  (0.79 ± 0.01) | 11.36  (11.34 ± 0.04) | 10.47 | 57.29  (54.55 ± 1.80) | 5.16  (4.91 ± 0.17) |
| 1.0:1.2 | - | 0.77  (0.77 ± 0.01) | 12.09  (11.98 ± 0.16) | 11.41 | 62.37  (62.13 ± 0.46) | 5.83  (5.77 ± 0.05) |
|  | 1 vol % | 0.77  (0.77 ± 0.01) | 12.68  (12.24 ± 0.33) | 11.36 | 63.24  (63.15 ± 0.39) | 6.16  (5.97 ± 0.13) |
|  | 3 vol % | 0.77  (0.77 ± 0.00) | 11.68  (11.62 ± 0.18) | 11.72 | 63.36  (62.61 ± 0.92) | 5.72  (5.64 ± 0.10) |
| 1.0:1.5 | - | 0.78  (0.77 ± 0.00) | 11.29  (11.41 ± 0.12) | 11.25 | 64.94  (63.51 ± 1.14) | 5.72  (5.61 ± 0.11) |
| 1.0:2.0 | - | 0.79  (0.78 ± 0.01) | 11.02  (11.25 ± 0.20) | 10.17 | 60.84  (58.91 ± 1.97) | 5.29  (5.16 ± 0.18) |


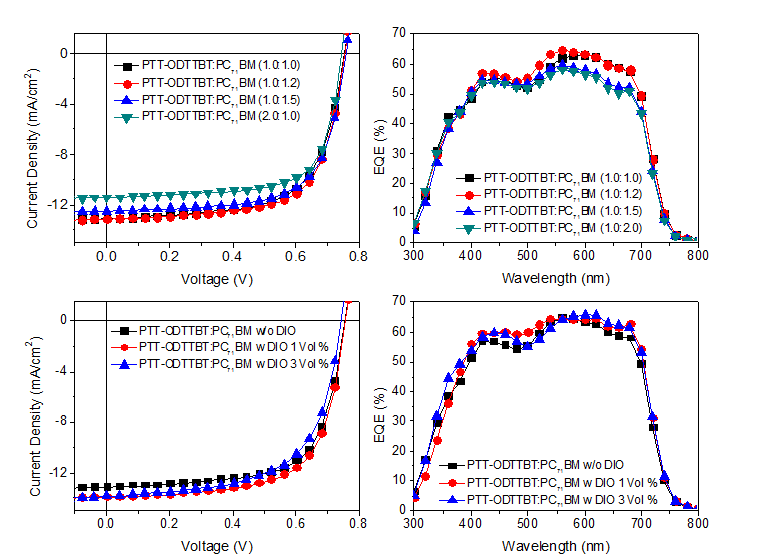


**Figure S7.** *J-V* characteristics and EQE curves of the OPV devices based on PTT-ODTTBT:PC_71_BM with various blending ratios (up) and DIO volumes (down).

**Table S2.** Performances of the OPV devices based on PTT-ODTTBT:PC_71_BM.

| **Ratio** | **DIO** | ***V*_oc_**  **(V)** | ***J*_sc_**  **(mA/cm^2^)** | ***J*_sc,calc._**  **(mA/cm^2^)** | ***FF***  **(%)** | ***PCE***  **(%)** |
| --- | --- | --- | --- | --- | --- | --- |
| 1.0:1.0 | - | 0.75  (0.75 ± 0.00) | 13.09  (12.92 ± 0.16) | 12.48 | 65.45  (65.20 ± 0.41) | 6.43  (6.35 ± 0.05) |
| 1.0:1.2 | - | 0.75  (0.76 ± 0.01) | 13.13  (12.93 ± 0.18) | 12.75 | 67.88  (68.19 ± 0.38) | 6.70  (6.67 ± 0.04) |
|  | 1 vol % | 0.75  (0.75 ± 0.00) | 13.96  (13.83 ± 0.11) | 13.32 | 66.94  (66.47 ± 0.63) | 7.05  (6.88 ± 0.12) |
|  | 3 vol % | 0.74  (0.74 ± 0.00) | 13.85  (13.82 ± 0.14) | 13.25 | 62.17  (61.87 ± 0.33) | 6.38  (6.34 ± 0.04) |
| 1.0:1.5 | - | 0.76  (0.75 ± 0.01) | 12.50  (12.39 ± 0.10) | 11.80 | 68.01  (67.48 ± 2.08) | 6.43  (6.27 ± 0.17 |
| 1.0:2.0 | - | 0.74  (0.73 ± 0.01) | 11.40  (11.64 ± 0.23) | 11.53 | 70.58  (68.45 ± 1.47) | 5.97  (5.85 ± 0.07) |

**Table S3.** Azimuthal angle scan profiles for the synthesized polymer films at the (100) peak.

| **Films** | **Azimuthal Angle (100)** | | | |
| --- | --- | --- | --- | --- |
|  | **PT-ODTTBT** | | **PTT-ODTTBT** | |
|  | **Edge on**  **(%)** | **Face on**  **(%)** | **Edge on**  **(%)** | **Face on**  **(%)** |
| Neat Polymer | 93.5 | 6.5 | 82.7 | 17.3 |
| Polymer:PC_71_BM | 87.7 | 12.3 | 74.5 | 25.5 |
| Polymer:PC_71_BM w DIO | 79.2 | 20.8 | 62.3 | 37.7 |
